# Circulating Lipid Peroxides Associate with More Aggressive Tumor Phenotypes and Increased Risks of Recurrence and Mortality in Breast Cancer Patients

**DOI:** 10.3390/medsci14010043

**Published:** 2026-01-16

**Authors:** Julia Fernandes Gois Orrutéa, Rafaela Oliveira Matos, João Paulo Araújo Pinto, André Cherubini Cechinel, Bruna Yukie Koizumi, Rafael Gomes Paz, Rafaella Frederico Almeida, Janaína Carla da Silva, Tatiane Renata Fagundes, Daniel Rech, Guilherme Welter Wendt, Carolina Panis

**Affiliations:** 1 Laboratory of Tumor Biology, Western Paraná State University, Francisco Beltrão 85605-010, PR, Brazil; 2 Departamente de Biochemistry and Molecular Medicine, Université de Montréal, Montreal, QC H3T 1J4, Canada; 3 Postgraduate Program in Applied Health Sciences, Western Paraná State University, Francisco Beltrão 85601-010, PR, Brazil

**Keywords:** lipid peroxidation, breast cancer, oxidative stress, prognostic biomarker

## Abstract

**Background/Objectives**: Breast cancer is the most common neoplasm among women and remains the leading cause of cancer-related mortality in the female population worldwide. Tumor cells exist within a highly oxidative microenvironment, which promotes the formation of substantial amounts of lipid peroxides. However, the clinical significance of circulating lipid peroxides in breast cancer is still not well understood. **Methods**: In this study, we quantified systemic lipid peroxide levels in plasma samples from 408 breast cancer patients and examined their associations with key clinicopathological parameters to evaluate their potential as disease biomarkers. Data are reported as relative light units (RLU). **Results**: Our findings revealed significantly higher lipid peroxide levels in HER2-amplified tumors compared with estrogen-receptor-positive tumors (1,133,494 ± 102,409 RLU vs. 951,883 ± 47,535 RLU; *p* = 0.0438). Elevated levels were also observed in patients with triple-negative breast cancer relative to those with Luminal A (1,163,323 ± 109,640 RLU vs. 875,633 ± 49,601 RLU; *p* = 0.0356) and Luminal B tumors (1,163,323 ± 109,640 RLU vs. 1,071,779 ± 98,329 RLU; *p* = 0.0254). In addition, increased lipid peroxidation was detected in patients with high-grade tumors (G3: 1,141,035 ± 101,045 RLU vs. G1–G2: 949,658 ± 46,119 RLU; *p* = 0.0346) and in those classified as at high risk of recurrence or death compared with low-risk patients (1,209,530 ± 95,396 RLU vs. 978,318 ± 229,526 RLU; *p* = 0.0054). Overweight patients also exhibited higher lipid peroxide levels than eutrophic individuals (1,131,233 ± 59,633 RLU vs. 820,772 ± 57,653 RLU; *p* = 0.0142). **Conclusions**: Collectively, these results suggest that circulating lipid peroxides may serve as potential biomarkers for recurrence and death risk in breast cancer, particularly among patients with more aggressive tumor phenotypes.

## 1. Introduction

Breast cancer is the most frequently diagnosed malignancy and a leading cause of cancer-related death among women worldwide [1]. This multifactorial and heterogeneous disease is strongly influenced by inflammatory processes, which play a critical role from early carcinogenesis to tumor dissemination [2]. Oxidative stress is a key biological event in this context, arising when the production of reactive oxygen and nitrogen species (RS) exceeds the capacity of antioxidant defenses [3]. This redox imbalance promotes DNA damage, lipid peroxidation, protein oxidation, and dysregulation of signaling pathways, thereby contributing to genomic instability, inflammation, and a tumor-promoting microenvironment closely linked to breast carcinogenesis and disease progression [4].

RS readily interact with several cellular components, particularly membrane lipids, generating lipid peroxides. These molecules have been widely investigated as potential disease markers, as their concentrations vary significantly across clinical outcomes in breast cancer. Elevated lipid peroxide levels have been documented in breast tumor tissue compared with normal mammary tissue, suggesting that tumors may serve as a major source of these molecules and modulate the surrounding microenvironment by altering local oxidative balance [5]. High levels of lipid peroxides have also been reported in post-menopausal young patients [6], as well as in the plasma of individuals with triple-negative breast cancer [7]. Moreover, patients with lymph node metastasis demonstrate increased lipid peroxide levels, indicating their potential relevance for assessing disease extent [8].

Lipid peroxides are additionally influenced by breast cancer treatment. Chemotherapy has been shown to increase systemic lipid peroxidation in breast cancer patients [9]. Conversely, patients with advanced-stage disease receiving paclitaxel or doxorubicin often present increased lipid peroxide levels, which have been associated with treatment-related toxicity [10]. Environmental exposures, such as pesticides, can further modulate lipid peroxidation in breast cancer patients and have been linked to worse clinical outcomes.

Despite these findings, the relevance of lipid peroxides as markers in predicting breast cancer recurrence and mortality remains poorly explored, especially in patients who have not yet started antitumor treatment, since these molecules can shape the tumor microenvironment and support breast cancer growth and spread. Therefore, this study evaluated a large cohort of breast cancer patients at the time of diagnosis, before any chemotherapy, and quantified plasma lipid peroxide levels using high-sensitivity chemiluminescence to examine their associations with clinicopathological characteristics related to the risk of recurrence and death.

## 2. Materials and Methods

This was a mixed exploratory study, including both retrospective and prospective components, involving the analysis of peripheral blood plasma samples from breast cancer patients treated at Associação Beneficente Deus Menino (ABDM)—Hospital de Cancer de Francisco Beltrão (Ceonc), Paraná, Brazil, between April 2015 and August 2024. The study was approved by the Institutional Research Ethics Committee and registered in Plataforma Brasil (CAAE 35524814.4.0000.0107, date of approval 25 September 2014). All participants provided written informed consent. All samples were collected prior to the initiation of any chemotherapy regimen.

After screening 998 patients, a total of 408 individuals were included in the study based on sample quality, analytical criteria, and availability of clinicopathological and medical record data. Clinical variables collected comprised age at diagnosis, tumor histopathology, Ki-67 proliferation index, estrogen and progesterone receptor (ER/PR) status, human epidermal growth factor 2 receptor amplification status (HER2), molecular subtype, tumor size, histological grade, angiolymphatic invasion, lymph node and distant metastases, body mass index (BMI), menopausal status, treatment response, recurrence, and mortality.

Molecular subtyping was performed using immunohistochemistry for estrogen (ER), progesterone (PR), Ki-67 index, and HER2 amplification. Tumors were classified according to St. Gallen criteria [11] as Luminal A (ER/PR+, HER2–, Ki-67 < 14%), Luminal B (ER/PR+, HER2–, Ki-67 > 14%), HER2-enriched (HER2 3+ or 2+ with ISH confirmation, any Ki-67), and Triple Negative (ER–/PR–/HER2–, any Ki-67).

Risk stratification for disease recurrence and mortality was established using a comprehensive set of clinical and pathological variables obtained from patient medical records. The specific clinical information used for this purpose is detailed in Table 1. Patients were categorized into low-, intermediate-, and high-risk groups.

According to these criteria, low-risk patients were those with negative lymph nodes and all of the following: tumor size under 2 cm, histological grade 1, ER or PR positivity, HER2 negativity, luminal A molecular subtype, and age ≥ 35 years. Intermediate-risk included patients with negative lymph nodes but at least one unfavorable feature—tumor size > 2 cm, histological grade 2–3, ER or PR negativity, luminal B/HER2-negative subtype, or age < 35 years—or patients with 1 to 3 positive lymph nodes who remained ER/PR-positive. High-risk patients were defined as those with four or more positive lymph nodes, or lymph node-negative tumors presenting ER-/PR-/HER2- profiles and tumor size > 2 cm, or lymph node-negative tumors larger than 1 cm with HER2 positivity. Patients who died during the five-year follow-up period are referred to as deceased. All biological samples used for lipid peroxide determination were collected at baseline, prior to treatment initiation. Therefore, death occurred after sample collection and during the follow-up period.

Treatment response was evaluated in early-stage breast cancer patients monitored for disease recurrence who received neoadjuvant or adjuvant chemotherapy. Tumor response was assessed according to RECIST version 1.1 by an oncologist, based on a comprehensive review of patients’ medical records and clinical data throughout the follow-up period. Baseline and follow-up tumor evaluations were performed using imaging modalities recommended by RECIST, including mammography, ultrasound at diagnosis, breast magnetic resonance imaging, computed tomography, and positron emission tomography–computed tomography during follow-up. For each patient, the same imaging modality was consistently used to compare initial and final assessments. Treatment response was categorized as complete response (defined as the disappearance of all target lesions), partial response (a reduction of at least 30% in the sum of target lesion diameters without the emergence of new lesions), progressive disease (an increase of at least 20% in lesion size relative to baseline and/or the appearance of new lesions in the breast or distant sites), or stable disease (insufficient change to meet criteria for partial response or progressive disease). Patients were followed for a total period of five years. Based on these criteria, individuals achieving a complete response were classified as responsive, whereas those with partial response, stable disease, or progressive disease were classified as chemoresistant [12].

Lipid peroxide levels were measured using a chemiluminescence-based method [13], previously validated in studies by our group [14,15]. All chemicals were provided from Sigma Aldrich, St. Louis, MO, USA. Tert-butyl hydroperoxide was used as a strong peroxyl radical inducer, initiating lipid peroxidation by attacking membrane lipids [16]. This reaction generates photon emission detectable by chemiluminescence. Higher antioxidant concentrations prolong the induction period before the onset of lipid peroxidation. Heparinized peripheral blood (10 mL) was collected preoperatively and centrifuged (5 min, 4000 rpm). Plasma was aliquoted and stored at −20 °C. For analysis, 125 μL of plasma was mixed with 855 μL of monobasic phosphate buffer (NaH_2_PO_4_, 30 mM, pH 7.4) at 37 °C, followed by the addition of 20 μL tert-butyl hydroperoxide solution (3 mM). Chemiluminescence was recorded using a luminometer (Glomax, Promega^®^, Madison, WI, USA) with a 2400 s protocol (1 read/s). Results were reported as the total relative light units (RLU) obtained from integration of the total emission curve. Samples were analyzed in duplicate, using the same reagent lots, and not subjected to freeze–thaw cycles. Intra-assay variability was minimized by strict control of sample handling, temperature, and timing, following a standardized protocol with fixed incubation times. Inter-assay variability was monitored using internal quality control samples, which showed consistent performance across samples. No batch effects were observed since internal controls did not show significant variation across measurements. Thus, normalization for batch effects was not required.

Data analysis was carried out in GraphPad Prism 9.0, Microsoft Excel 2016, and OriginLab 9.0, including normality, non-parametric tests and ROC curve analysis. Thus, comparisons were performed using the Mann–Whitney test for two groups, and one-way ANOVA followed by Kruskal–Wallis and Dunn’s test for three or more groups. The ROC analysis included 412 women diagnosed with cancer and 586 controls without a cancer diagnosis. Control participants were recruited from the same geographic region and during the same study period as the cancer cases. Controls were age- and menopausal-status–matched to cancer cases in order to minimize confounding effects related to these variables, which are known to influence oxidative stress. The ROC analysis was designed to assess the overall discriminatory capacity of the oxidative stress marker between cancer and non-cancer groups. For box plot graphs, error bars indicate the minimum and maximum ranges, while the boxes represent the median values and their dispersion within each group. Statistical significance was defined as *p* < 0.05, and data are presented as mean ± standard error of the mean (SEM). Fold changes were calculated as the ratio between the median biomarker levels for each group comparison.

## 3. Results

Among the 408 samples analyzed, most patients exhibited ER positivity (71.3%) and PR positivity (50.0%), along with elevated proliferative activity indicated by Ki-67 ≥ 14% (61.5%). The predominant molecular subtype was Luminal B (37.0%), and more than half of the cohort fell into the intermediate risk category for recurrence or death (52.4%). Tumor size greater than 2 cm was observed in 51.2% of cases, and the majority of tumors were classified as low or intermediate histological grade (74.3%). Demographically, most women were aged 50 years or older at diagnosis (61.5%), half were postmenopausal (50.7%), and more than half were overweight or obese (56.9%). The overall clinicopathological characteristics of patients diagnosed with breast cancer are summarized in Table 2. Patients with missing information (NR) were excluded from risk stratification analysis.

**Figure 1:**
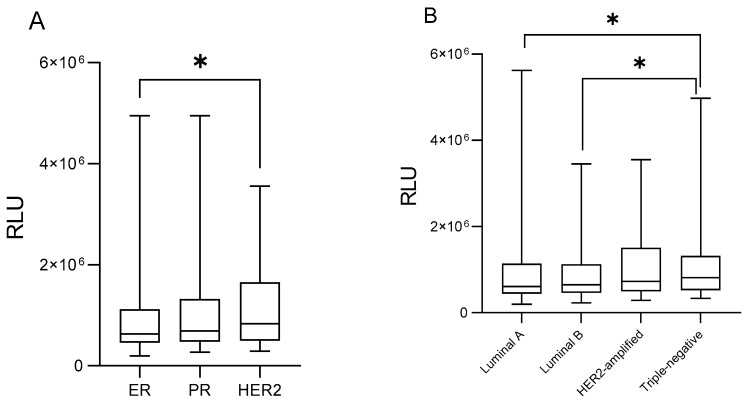
Plasma levels of lipid peroxides in breast cancer patients according to tumor receptor status (**A**) and its molecular subtype classification (**B**). Error bars indicate the minimum and maximum ranges, while the boxes depict the median values and their dispersion for each group. Asterisks (*) denote statistically significant differences (*p* < 0.05). RLU = relative light units. ER = estrogen receptor positivity, PR = progesterone receptor positivity, HER2 = positive for HER2 amplification.

Figure 1A depicts lipid peroxidation levels in ER-positive, PR-positive, and HER2-amplified tumors, respectively. Lipid peroxide levels were significantly higher in HER2-positive tumors compared to ER-positive tumors (1,133,494 ± 102,409 RLU vs. 951,883 ± 47,535 RLU; *p* = 0.0438; Figure 1A, 1.20-foldchange). Figure 1B illustrates lipid peroxidation profiles according to breast cancer molecular subtypes. Patients having triple-negative tumors exhibited increased levels of lipid peroxides relative to both Luminal A (1,163,323 ± 109,640 RLU vs. 1,071,779 ± 98,329 RLU, *p* = 0.0356, 1.10-foldchange) and Luminal B tumors (1,163,323 ± 109,640 RLU vs. 875,633 ± 49,601 RLU; *p* = 0.0254, Figure 1B, 1.33-foldchange).

**Figure 2:**
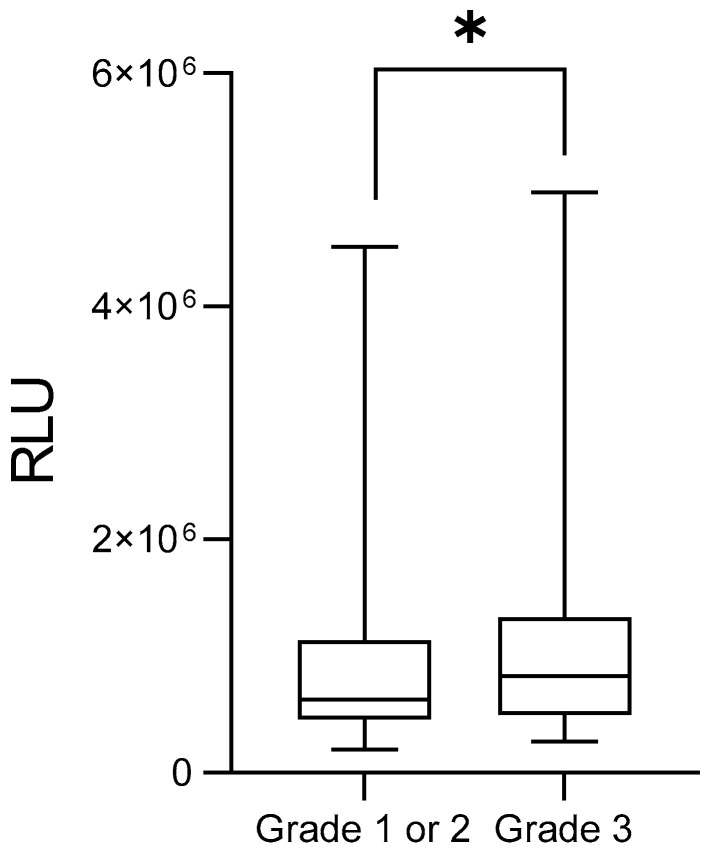
Plasma levels of lipid peroxides in breast cancer patients according to tumor histological grade. Error bars indicate the minimum and maximum ranges, while the boxes depict the median values and their dispersion for each group. Asterisks (*) denote statistically significant differences (*p* < 0.05). RLU = relative light units.

With regard to histological grade, patients carrying grade 3 tumors showed markedly higher lipid peroxidation compared with grade 1 or 2 tumors (G3: 1,141,035 ± 101,045 RLU vs. G1/2: 949,658 ± 46,119 RLU; *p* = 0.0346; Figure 2, 1.20-foldchange).

**Figure 3:**
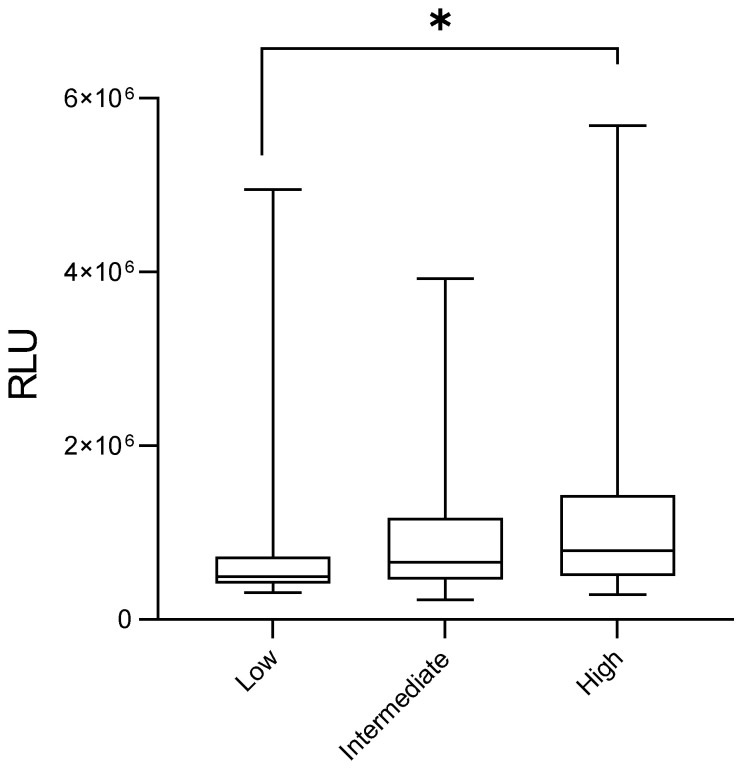
Plasma levels of lipid peroxides in breast cancer patients according to disease recurrence and death risk stratification. Error bars indicate the minimum and maximum ranges, while the boxes depict the median values and their dispersion for each group. Asterisks (*) denote statistically significant differences (*p* < 0.05). RLU = relative light units.

**Figure 4:**
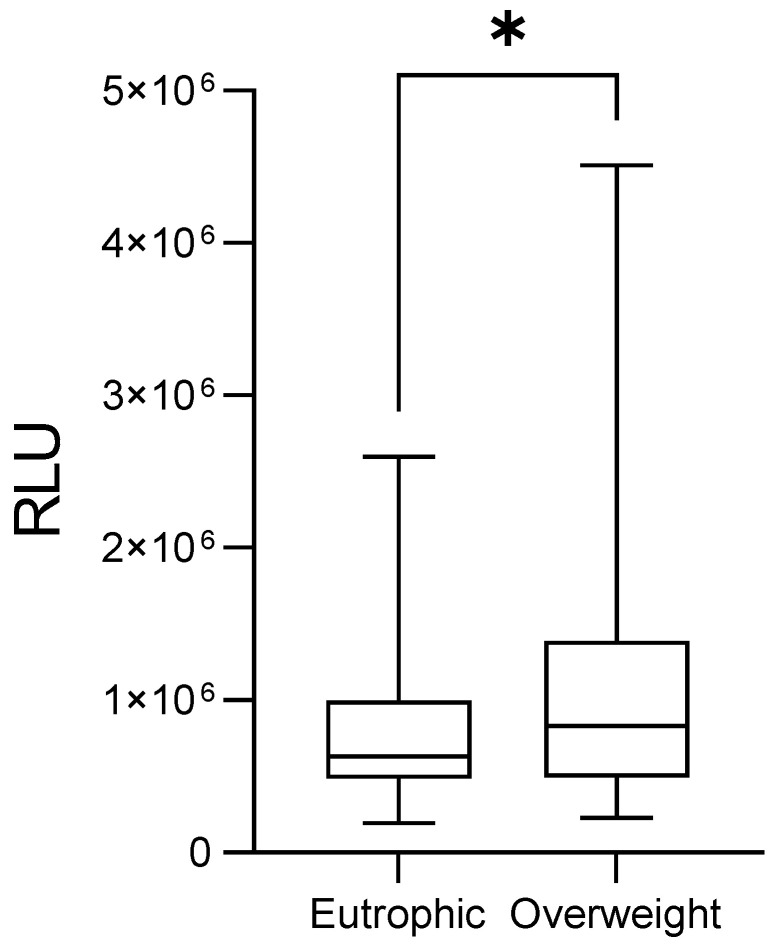
Plasma levels of lipid peroxides in breast cancer patients according to their body mass index. Error bars indicate the minimum and maximum ranges, while the boxes depict the median values and their dispersion for each group. Asterisks (*) denote statistically significant differences (*p* < 0.05). RLU = relative light units.

Additionally, patients classified as high-risk for recurrence or death presented significantly elevated peroxide levels compared to those at low risk (1,209,530 ± 95,396 RLU vs. 978,318 ± 229,526 RLU; *p* = 0.0054; Figure 3, 1.24-foldchange). Also, overweight patients displayed higher levels than eutrophic patients (1,131,233 ± 59,633 RLU vs. 820,772 ± 57,653 RLU; *p* = 0.0142, Figure 4, 1.38-foldchange).

**Figure 5:**
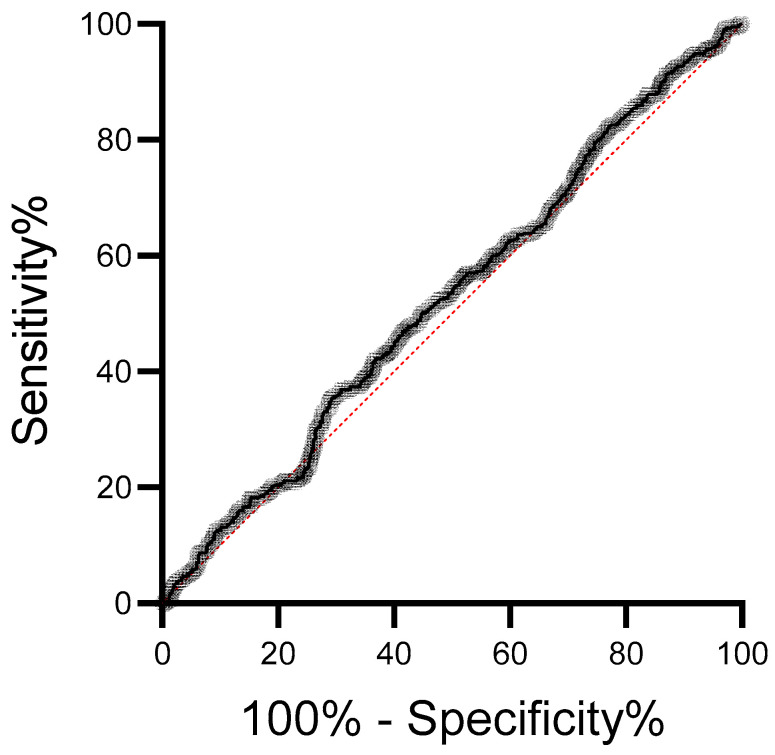
Receiver operating characteristic (ROC) curve evaluating the performance of plasma lipid peroxide levels in distinguishing breast cancer patients from healthy controls. The ROC plot displays sensitivity (%) against 100%—specificity (%) for all possible cutoff values. The black line represents the empirical ROC curve, and the red dashed line indicates the reference diagonal corresponding to random classification.

The ROC curve analysis (Figure 5) demonstrated that plasma lipoperoxide levels showed limited discriminatory ability between healthy controls (*n* = 586) and breast cancer patients (*n* = 408). The area under the curve (AUC) was 0.5262, with a standard error of 0.01850 and a 95% confidence interval ranging from 0.4900 to 0.5625. The AUC did not differ significantly from random classification (*p* = 0.1577).

## 4. Discussion

Lipid peroxides are among the most widely explored oxidative stress markers in cancer, yet their clinical significance remains far from clearly established. In this study, we demonstrated that circulating levels of lipid peroxides vary according to the clinical presentation of breast cancer, and we observed higher levels in overweight patients and in those with more aggressive molecular subtypes, higher histological grades, and a greater risk of disease recurrence and mortality. These findings contribute to the existing literature by highlighting circulating lipid peroxides as potential biomarkers, particularly in the context of recurrence and death risk in breast cancer.

Previous studies have described substantial variability in systemic lipid peroxide levels, supporting their potential clinical relevance. In our cohort, HER2-positive tumors exhibited significantly higher lipid peroxide concentrations compared with ER-positive tumors. HER2—a transmembrane growth factor receptor—is strongly linked to aggressive tumor phenotypes and therapeutic resistance [17]. Patients with HER2 overexpression frequently display impaired antioxidant capacity, a condition that may help explain our findings and reinforce the hypothesis of a pro-inflammatory tumor microenvironment driven by HER2-associated signaling pathways [18].

In addition to its well-established role in cell proliferation and survival, evidence suggests that HER2 hyperactivation also contributes to redox imbalance by increasing mitochondrial activity and oxidative phosphorylation, resulting in excessive ROS generation. A mitochondrial pool of HER2, imported via mtHSP70, may further disrupt oxidative metabolism by increasing oxygen consumption, ATP production, and electron transport chain activity while simultaneously promoting glycolytic flux [19,20]. Additionally, dysfunctional mitochondria in HER2-positive cells may interact with NADPH oxidases, such as NOX1, thereby amplifying ROS production and reinforcing oncogenic signaling pathways [21]. Recent findings also indicate that HER2-positive tumors exhibit altered expression patterns of redox-related genes, which could influence immune cell composition within the tumor microenvironment and further link HER2 signaling to oxidative stress and immune dysregulation [22].

We also found that patients with triple-negative breast cancer (TNBC) displayed more pronounced lipid peroxidation compared to those with Luminal A and B subtypes. TNBC lacks classical therapeutic targets (ER, PR, and HER2), resulting in more aggressive behavior and fewer treatment options [23]. TNBC cell lines exhibit impaired mitochondrial respiration and elevated mitochondrial ROS production [24]. Comparative analyses have shown that certain TNBC cell lines generate significantly higher ROS levels than receptor-positive counterparts, with mitochondria identified as the major source. Elevated ROS appear essential for sustaining oncogenic signaling in TNBC, as antioxidant treatments preferentially induce cell death in these models [25]. In humans, studies suggest that TNBC cells produce high mitochondrial ROS and secrete cytokines that recruit tumor-associated macrophages (TAMs), thereby amplifying oxidative stress in the tumor microenvironment [25,26]. Overall, the metabolic plasticity characteristic of TNBC allows both enhanced proliferation and adaptive redox responses, contributing to the poor prognosis associated with this subtype.

Enhanced lipid peroxidation was also observed in patients with high-grade tumors. This finding is clinically relevant, as high-grade tumors typically consist of poorly differentiated cells with increased proliferative, invasive, and metastatic potential [27,28]. These results suggest a mechanistic link between oxidative stress and the adverse outcomes associated with high-grade malignancies. Notably, HER2-positive and TNBC tumors—commonly represented within high-grade categories—harbor intrinsic mechanisms that disrupt redox homeostasis, further intensifying oxidative stress in the tumor microenvironment [27]. Excessive ROS generation not only reflects oxidative burden but can actively drive oncogenic signaling by modulating pathways such as PI3K/Akt, JAK/STAT, MAPK, NF-κB, and Nrf2, creating a hostile biochemical environment that promotes inflammation, sustains proliferation, invasion, angiogenesis and tumor progression [29,30,31].

A central finding of our study is the observed difference in lipid peroxide levels between patients classified as low versus high recurrence and mortality risk. To our knowledge, this is the first study to report such an association in this context. Patients in the low-risk group displayed lower lipid peroxidation compared with those in the high-risk category. Although we did not observe significant differences in lipid peroxide levels in survival profiles of the patients, this marker appears relevant as a potential indicator of breast cancer recurrence and mortality when measured at diagnosis, suggesting that its assessment over the course of patient follow-up may have clinical value in future studies. Breast cancer risk stratification is a fundamental for guiding adjuvant therapy and integrates clinical and pathological characteristics, including age, lymph node status, and molecular subtype, with more stringent recommendations for adjuvant chemotherapy as recurrence risk increases [32]. Supporting our observations, Saintot et al. [33] reported that patients with elevated plasma lipid peroxides were more likely to experience recurrence. Reactive oxygen species (ROS) are proposed to act as intermediates that enhance neoplastic progression, activate proliferative signaling, and promote invasiveness, which may underlie the elevated lipid peroxidation observed in higher-risk groups [29].

Obese patients in our cohort exhibited higher lipid peroxidation, likely reflecting the metabolically dysregulated and pro-inflammatory environment characteristic of excessive adiposity. Expanded adipose tissue secretes pro-inflammatory cytokines such as TNF-α, IL-1β, and IL-6, which stimulate ROS production and activate NF-κB and NADPH oxidase pathways [34,35,36]. Elevated circulating free fatty acids may further exacerbate oxidative stress by fueling mitochondrial and peroxisomal ROS generation [37]. Additionally, adipose expansion promotes macrophage infiltration and chronic inflammation, processes that synergize with insulin resistance and hyperglycemia to intensify ROS production and sustain oxidative stress in the tumor microenvironment [38]. The increased cancer risk observed in insulin-resistant individuals may also stem from ROS-mediated DNA damage, promoting mutagenesis and carcinogenesis [39]. Together, these mechanisms appear to converge into a redox-imbalanced, pro-carcinogenic milieu that not only elevates lipid peroxidation but may also contribute to poorer outcomes in obese breast cancer patients.

This study presents some limitations. General health status, comorbidities, and treatments may have influenced the findings. Moreover, the five-year follow-up may not fully capture late disease recurrence, particularly in ER-positive breast cancer. ROC curve analysis indicated that plasma lipid peroxides have poor diagnostic value for distinguishing breast cancer patients from healthy controls. The AUC value near 0.5, coupled with lack of statistical significance, suggests that lipid peroxides alone cannot capture the complex oxidative and inflammatory processes underlying breast carcinogenesis. Also, although controls were matched to cancer cases by age and menopausal status, other factors that may influence oxidative stress levels, such as body mass index (BMI), lifestyle, and metabolic conditions, were not included as matching or adjustment variables. Therefore, residual confounding cannot be completely excluded and should be considered when interpreting the ROC analysis. Further, although subgroup analyses were performed, the sample sizes within these strata were insufficient to support reliable multivariable analyses. Consequently, adjustment for potential confounders was not feasible, and the observed associations should be interpreted with caution and considered exploratory. Finally, the absence of inflammatory marker assessment represents an additional limitation of this study, as it restricts a more comprehensive evaluation of lipid peroxidation markers as potential biomarkers in breast cancer. Despite this, our findings indicate that elevated lipid peroxide levels may serve as meaningful prognostic biomarkers in breast cancer, particularly in relation to more aggressive breast cancer and recurrence and mortality risk stratification.

## 5. Conclusions

The results of this work open new perspectives for the use of lipid peroxides as biomarkers in breast cancer. Circulating levels of lipid peroxides can serve as valuable tools to assist physicians in patient prognosis and therapeutic decision-making, ultimately contributing to more effective treatment strategies and a reduction in adverse clinical outcomes.

## Figures and Tables

**Table 1:** Parameters evaluated for risk stratification of recurrence and death of breast cancer patients.

	Negative lymph nodes and all the following criteria:
	pT under 2 cm;
Low risk	Histological grade 1;
	ER- or PR-positive;
	HER-2-negative;
	Molecular subtype luminal A; and
	Age equal or above 35 years old.
	Negative lymph nodes and at least one of the following criteria:
	pT higher than 2 cm; or
	Histological grade 2–3; or ER- or PR-negative; or
Intermediate risk	Molecular subtype luminal B (HER-2-negative); or
	Age under 35 years old; or yet
	1 to 3 affected lymph nodes. If ER- and PR-positive.
	4 or more positive lymph nodes; or
High risk	Lymph nodes negative with ER. PR- and HER-2-negative. pT higher than 2 cm; or
	Lymph node negative. pT higher than 1 cm and HER-2-positive.

**Table 2:** Clinicopathological profile of breast cancer patients included in the study.

Number of Breast Cancer Patients in the Study	*N* = 408
Estrogen receptor	
No	28.70%
Yes	71.30%
Progesterone receptor	
No	50.00%
Yes	50.00%
HER2 receptor	
No	83.60%
Yes	16.40%
Ki-67 proliferation index	
<14%	35.80%
≥14%	61.50%
NR	2.70%
Molecular subtype	
Luminal A	28.70%
Luminal B	37.00%
HER-2-amplified	13.50%
Triple-negative	18.90%
NR	1.90%
Risk of death/recurrence	
Low	7.40%
Intermediate	52.40%
High	31.90%
NR	8.30%
Tumor size	
≤2 cm	44.90%
>2 cm	51.20%
NR	3.90%
Histological grade	
Grades 1 and 2	74.30%
Grades 3	22.00%
NR	3.70%
Intratumoral emboli	
No	75.20%
Yes	21.30%
NR	3.50%
Lymph node metastasis	
No	66.20%
Yes	29.90%
NR	3.90%
Distant metastasis	
No	72.30%
Yes	6.40%
NR	21.30%
Age at diagnosis	
<50 years	33.30%
≥50 years	61.50%
NR	5.20%
Menopause at diagnosis	
No	41.40%
Yes	50.70%
NR	7.90%
Body Mass Index (BMI)	
Eutrophic	21.10%
Overweight	56.90%
NR	22%
Chemoresistance	
No	83.10%
Yes	16.90%
Recurrence	
No	90.20%
Yes	9.80%
Deceased	
No	94.40%
Yes	5.60%

## Data Availability

The data presented in this study are available on request from the corresponding author due to privacy.

## Abbreviations

The following abbreviations are used in this manuscript:

ABDM	Associação Beneficente Deus Menino
BMI	Body Mass Index
ER	Estrogen Receptor
EU	Eutrophic
G1	Histological Grade 1
G2	Histological Grade 2
G3	Histological Grade 3
HER2	Human Epidermal Growth Factor Receptor 2
HR	High Risk
LA	Luminal A
LB	Luminal B
LR	Low Risk
NR	Not reported
OW/OB	Overweight/Obese
PR	Progesterone Receptor
ROS	Reactive Oxygen Species
RLU	Relative Light Unit
TAMs	Tumor-associated macrophages
TN	Triple Negative
TNBC	Triple-negative breast cancer
